# Plasmid Characterization and Chromosome Analysis of Two *netF*+ *Clostridium perfringens* Isolates Associated with Foal and Canine Necrotizing Enteritis

**DOI:** 10.1371/journal.pone.0148344

**Published:** 2016-02-09

**Authors:** Iman Mehdizadeh Gohari, Andrew M. Kropinski, Scott J. Weese, Valeria R. Parreira, Ashley E. Whitehead, Patrick Boerlin, John F. Prescott

**Affiliations:** 1 Department of Pathobiology, University of Guelph, Guelph, Ontario N1G 2W1, Canada; 2 Department of Veterinary Clinical and Diagnostic Sciences, University of Calgary, Calgary, Alberta, CSB 101E, Canada; Institute Pasteur, FRANCE

## Abstract

The recent discovery of a novel beta-pore-forming toxin, NetF, which is strongly associated with canine and foal necrotizing enteritis should improve our understanding of the role of type A *Clostridium perfringens* associated disease in these animals. The current study presents the complete genome sequence of two *netF*-positive strains, JFP55 and JFP838, which were recovered from cases of foal necrotizing enteritis and canine hemorrhagic gastroenteritis, respectively. Genome sequencing was done using Single Molecule, Real-Time (SMRT) technology-PacBio and Illumina Hiseq2000. The JFP55 and JFP838 genomes include a single 3.34 Mb and 3.53 Mb chromosome, respectively, and both genomes include five circular plasmids. Plasmid annotation revealed that three plasmids were shared by the two newly sequenced genomes, including a NetF/NetE toxins-encoding *tcp*-conjugative plasmid, a CPE/CPB2 toxins-encoding *tcp*-conjugative plasmid and a putative bacteriocin-encoding plasmid. The putative beta-pore-forming toxin genes, *netF*, *netE* and *netG*, were located in unique pathogenicity loci on *tcp*-conjugative plasmids. The *C*. *perfringens* JFP55 chromosome carries 2,825 protein-coding genes whereas the chromosome of JFP838 contains 3,014 protein-encoding genes. Comparison of these two chromosomes with three available reference *C*. *perfringens* chromosome sequences identified 48 (~247 kb) and 81 (~430 kb) regions unique to JFP55 and JFP838, respectively. Some of these divergent genomic regions in both chromosomes are phage- and plasmid-related segments. Sixteen of these unique chromosomal regions (~69 kb) were shared between the two isolates. Five of these shared regions formed a mosaic of plasmid-integrated segments, suggesting that these elements were acquired early in a clonal lineage of *netF*-positive *C*. *perfringens* strains. These results provide significant insight into the basis of canine and foal necrotizing enteritis and are the first to demonstrate that *netF* resides on a large and unique plasmid-encoded locus.

## Introduction

*Clostridium perfringens* is the best-known and most commonly isolated clostridial species [1]. Although *C*. *perfringens* is part of the normal intestinal flora and the majority of strains seem to be non-pathogenic, some are well recognized as being able to cause diseases in both animals and humans, ranging from myonecrosis and food poisoning to enterotoxemia and enteritis [1,2]. The pathogenicity of *C*. *perfringens* is directly attributable to the many toxins and extracellular enzymes that it produces [3–5]. The current typing system for *C*. *perfringens* (types A to E) is based on the major toxin production profile [1].

*Clostridium perfringens* type A-associated diarrheal and enteric disease in foals and dogs is not well characterized, and its understanding is complicated by the common presence of these bacteria in the intestinal tract and feces of healthy animals. However, recently, our group described three novel putative toxin genes encoding proteins related to the pore-forming Leukocidin/Hemolysin Superfamily; these were designated *netE*, *netF*, and *netG*. NetF has been implicated as the primary virulence factor of foal necrotizing enteritis and canine hemorrhagic gastroenteritis [6]. PFGE showed that canine and equine NetF-producing strains belong to a single clonal lineage. All *netF*-positive *C*. *perfringens* strains consistently carry two conjugative plasmids; one encoding *netF* and *netE*, and the other encoding *cpe*. A NetG toxin-encoding plasmid was only found in half of *netF*-positive strains [6].

Genome sequencing and comparative analyses have shown that *C*. *perfringens* share highly conserved backbone regions on the chromosome and that most of the major *C*. *perfringens* toxins are located on a family of *tcp*-conjugative plasmids [7–10]. Recognition that major *C*. *perfringens* toxins were plasmid-borne was a paradigm shift in understanding the basis of virulence in this bacterium. It is now well established that the genes encoding BEC (Binary enterotoxin), CPB, CPB2, ETX, ITX, NetF, NetB, TpeL, and sometime CPE are located on plasmids [6,11–13].

Almost all toxin plasmids [9,12,14–16] and some tetracycline resistance plasmids [17,18] are conjugative. These plasmids encode the *tcp* (Transfer of Clostridia Plasmids) locus, which shares minor sequence relatedness with the Tn916 conjugative transposon family [19]. The *tcp* locus encodes 11 conjugation proteins (IntP, TcpA to TcpJ), of which TcpA, TcpF, TcpG, TcpH are critical for conjugative transfer [19–21].

A comparative analysis of *tcp*-conjugative plasmids has shown that these plasmids share a highly conserved 35 kb core region and a diverse variable region. The core region is generally responsible for replication, plasmid maintenance/stability and conjugative transfer, and the variable region contains unique genes that are important for virulence of *C*. *perfringens* strains [9,12]. A general feature of *C*. *perfringens* toxin-carrying plasmids is the location of many toxin genes on pathogenicity loci (PaLoc) close to the DNA cytosine-methyltransferase (*dcm*) region, an insertional hot-spot for the mobile genetic elements that encode the toxin genes [9,12,22].

The present study describes the complete genome sequence of two *netF*-positive *C*. *perfringens* strains, JFP55 and JFP838, recovered from cases of foal necrotizing enteritis and canine haemorrhagic gastroenteritis, respectively. The particular emphasis of the current study is on the plasmids shared by these two *netF*-positive strains.

## Materials and Methods

### Bacterial Isolates and Genomic DNA Isolation

Two *netF*-positive type A *C*. *perfringens* strains, JFP55 and JFP838, recovered from cases of foal necrotizing enteritis and canine hemorrhagic gastroenteritis, respectively, were used in this study [6]. These isolates were selected on the basis of their clonal relationship identified in a previous study [6].

The genomic DNA of the samples was extracted using a modified version of the Qiagen bacterial DNA extraction protocol (Qiagen, Limburg, Netherlands) [23]. The quality of the genomic DNA was evaluated by standard agarose gel electrophoresis and the identity as the correct bacterium confirmed by PCR amplification of *cpa*, *cpe*, *netE*, *netF*, and *netG*.

### Genome Sequencing, Assembly and Annotation

Genome sequencing was performed by the McGill University and Génome Québec Innovation Centre (Montreal, QC, Canada). Two sequencing technologies, Single Molecule, Real-Time (SMRT) technology-PacBio and Illumina Hiseq2000 PE100 were used for both samples. This strategy provided an opportunity to successfully close the genome sequences of these two samples and the necessary accuracy of base calls for the sequences. *De novo* assembly was done using DNASTAR’s SeqMan NGen12 software (DNASTAR, Inc., Wisconsin, USA). Assembly errors and poor quality data were manually trimmed where possible. The contigs were oriented and ordered according to the closed *C*. *perfringens* chromosome ATCC13124 (GenBank Accession number NC_008261) using progressiveMauve alignment software [24]. Subsequently, the complete chromosome sequences of JFP55 and JFP838 were annotated by the Prokaryotic Genome Annotation Pipeline (http://www.ncbi.nlm.nih.gov/genomes/static/Pipeline.html).

Complete plasmid sequences of JFP55 and JFP838 were automatically annotated by MyRAST software, the next generation of Rapid Annotation using Subsystem Technology [25]. In addition, BLASTN and BLASTP analyzes [26] were performed to compare the query plasmid sequences with the NCBI database of known sequences.

The web-based server PHAST (**PHA**ge **S**earch **T**ool) [27] was used to identify the prophage sequences within the sequenced genomes.

### Identification of Unique Nucleotide Sequence using PanSeq

The Novel Region Finder of PanSeq software (http://lfz.corefacility.ca/panseq/) with a 500 bp cutoff was used to identify the unique chromosomal and plasmid nucleotide sequence of JFP55 and JFP838. The chromosomal unique regions were identified by comparison to three complete *C*. *perfringens* chromosome sequences: ATCC13124, strain 13 (GenBank NC_003366), and SM101 (GenBank CP000312).

The unique regions of conjugative plasmids of JFP55 and JFP838 were determined by comparison to six complete *C*. *perfringens* conjugative-plasmids: pCW3 (GenBank NC_010937), pCP8533etx (GenBank NC_011412), pCPF5603 (GenBank NC_007773), pCPF4969 (Genbank NC_007772), pCPPB-1 (GenBank NC_015712), and pNetB-NE10 (GenBank NC_019688).

In addition, BLASTN and BLASTP were used to determine the unique regions that were common to the two newly sequenced *netF*-positive *C*. *perfringens* isolates. To assign the putative function of the predicted CDSs (coding DNA sequence), significant similarity was defined as having an E value less than 10^−20^ and covering at least 80% of an CDS’s length available in GenBank.

### Identification of Core Nucleotide Sequence of Plasmids using PanSeq

The Pan-genome Analyses of PanSeq software was used to determine the core sequence of conjugative plasmids of this study by comparison to the same six complete *C*. *perfringens* conjugative-plasmids mentioned above.

### Nucleotide Sequence Accession Numbers

The GenBank accession numbers for nucleotide chromosome sequence of JFP55 and JFP838 are CP010993 and CP010994, respectively. The GenBank accession numbers for plasmid sequences are CP013615 for pJFP838A, KT020842 for pJFP838B, CP013040 for pJFP838C, CP013039 for pJFP838D, CP013038 for pJFP838E, CP013041 for pJFP55F, CP013042 for pJFP55G, CP013043 for pJFP55H, CP013044 for pJFP55J, and CP013045 for pJFP55K.

## Results

### Genome Sequencing and Assembly

The PacBio-SMRT sequencing technology generated 28,808 and 41,887 reads, with a raw median read length of 4,745 and 5,657 bp, totalling 183,795,040 (55-fold coverage) and 252,053,328 (71-fold coverage) nucleotides for JFP55 and JFP838, respectively. Moreover using Illumina Hiseq2000 platform, the average coverage was 50x for JFP55 and 80x for JFP838.

The McGill University and Génome Québec Innovation Centre conducted the initial *de novo* assembly using the Hierarchical Genome Assembly Process (HGAP) protocol version 2.0 in SMRT Analysis version 2.2.0. For JFP55, this assembly produced 64 contigs (minimum contig length: 524 and maximum contig length: 2,526,541) whereas for JFP838 46 contigs (minimum contig length: 535 and maximum contig length: 3,111,738) were generated. The final *de novo* assembly was done using DNASTAR’s SeqMan NGen12 tool and the quality of genome sequences and assembles was improved using the data generated by Illumina Hiseq2000 PE100. Furthermore, the assembled chromosomes were compared to the closed chromosome of ATCC13124 using progressiveMauve software to assess the validity of the assemblies.

The genome assembly of JFP55 and JFP838 yielded a complete chromosome and five circular plasmids each. A summary of the genome assembly results is presented in Table 1.

**Table 1:** Summary of genome assembly results of two *netF*-positive *C*. *perfringens* strains.

Strain	Chromosome size (bp)	Plasmids				
		Plasmid 1/Size (bp)	Plasmid 2/Size (bp)	Plasmid 3/Size (bp)	Plasmid 4/Size (bp)	Plasmid 5/Size (bp)
JFP838	3,530,414	pJFP838A/404,512	pJFP838B/66,958	pJFP838C/72,750	pJFP838D/48,597	pJFP838E/14,657
JFP55	3,347,300	pJFP55F/72,549	pJFP55G/36,664	pJFP55H/58,447	pJFP55J/42,209	pJFP55K/14,060

### Plasmids Shared by the Two *netF*-Positive *C*. *perfringens* Strains

Plasmid annotation revealed that both *netF*-positive *C*. *perfringens* strains, JFP55 and JFP838, harbor three plasmids in common, including a NetF/NetE toxins-encoding plasmid (pJFP55F, pJFP838C), a CPE/CPB2 toxins-encoding plasmid (pJFP55G, pJFP838D), and a putative bacteriocin-encoding plasmid (pJFP55K and pJFP838E).

#### NetF/NetE Toxins-Encoding Plasmids

**Fig 1:**
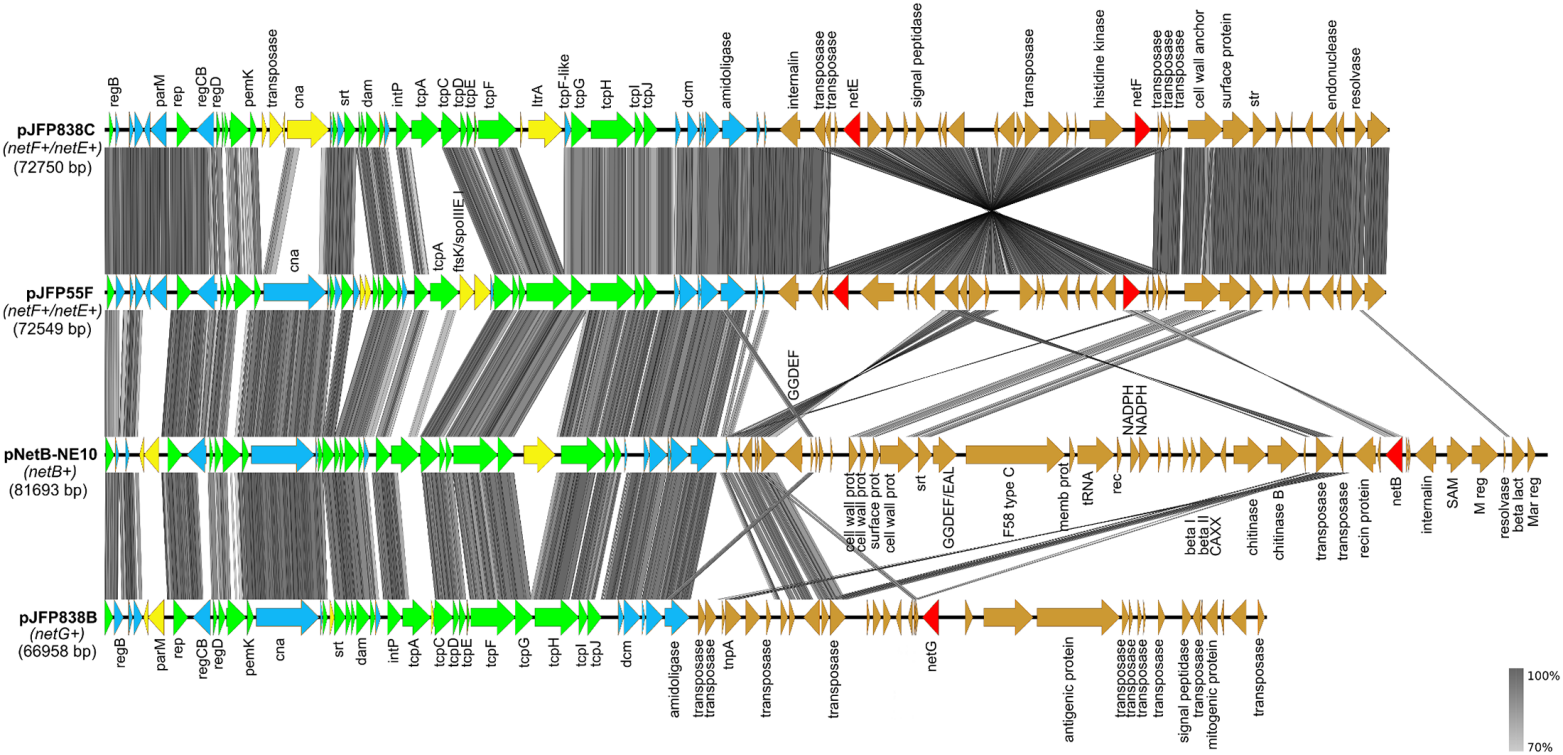
Comparative analysis of NetF/NetE toxins-encoding plasmids, NetB toxin-encoding plasmid and NetG toxin-encoding plasmid. Grey vertical blocks indicate the shared similarity regions according to TBLASTX identity. The coloured arrows represent CDSs; CDS arrows shown are as follows: green arrows, the conserved backbone CDSs shared by all *C*. *perfringens* conjugative plasmids; blue arrows, the other conserved CDSs shared by these four plasmids; yellow arrows, unique genes on each plasmid; red arrows, toxin genes; brown arrows, unique pathogenicity locus of each plasmid. The arrows with no name are hypothetical proteins.The image was generated using Easyfig [28].

The plasmids pJFP55F and pJFP838C are circular 72,549 bp and 72,750 bp plasmids with a G+C content of 25.37% and 25.39%, respectively. Sequence annotation of pJFP55F showed the presence of 79 CDSs whereas pJFP838C contained 82 CDSs. Sequence analysis with BLAST indicated that these plasmids, pJFP55F and pJFP838C, are highly similar (99% identity at DNA level) (Fig 1) and that they share 60% and 57% coverage, respectively, and within this common coverage, 95% sequence identity with plasmid pNetB-NE10 (Fig 1). The presence of *tcp* conjugation loci on pJFP55F and pJFP838C shows that these plasmids are members of *tcp*-conjugative family plasmids. Comparative analyses of NetF/NetE carrying plasmids with six *tcp*-conjugative plasmids, including pCW3 (tetracycline resistance-encoding plasmid), pCP8533etx (ETX/CPB2-encoding plasmid), pCPF5603 (CPE/CPB2-encoding plasmid), pCPF4969 (CPE-encoding plasmid), pCPPB-1 (CPE/ITX-encoding plasmid), and pNetB-NE10 (NetB-encoding plasmid) showed that these *tcp*-conjugative family plasmids share a highly conserved ~35 kb core region and possess a diverse variable region. The common backbone region contains 22 genes, which encode constituents of the *tcp* locus (*tcpACDEFGHIJ*), a plasmid replication gene (*rep*), a DNA-binding transcriptional repressor (*regD*), a growth inhibitor PemK protein, a sortase, a DNA adenine-specific methyltransferase (*dam*), a tyrosine site-specific recombinase, and seven hypothetical proteins with unknown functions. The common backbone genes identified in conjugative plasmids of JFP55 and JFP838 are listed in Table 2.

**Table 2:** Conserved core genome genes of *Clostridium perfringens tcp*-conjugative plasmids in JFP55 and JFP838.

NetF/NetE encoding plasmids		CPE/CPB2 encoding plasmids		NetG encoding plasmid	Product of gene
pJFP55F	pJFP838C	pJFP55G	pJFP838D	pJFP838B	
JFP55_pF0001	JFP838_pC0001	JFP55_pG0013	JFP838_pD0022	JFP838_pB0025	Hypothetical protein
JFP55_pF0007	JFP838_pC0007	JFP55_pG0007	JFP838_pD0016	JFP838_pB0031	Plasmid replication protein
JFP55_pF0009	JFP838_pC0009	JFP55_pG0005	JFP838_pD0014	JFP838_pB0033	DNA-binding transcriptional repressor
JFP55_pF0010	JFP838_pC0010	JFP55_pG0004	JFP838_pD0013	JFP838_pB0034	Hypothetical protein
JFP55_pF0011	JFP838_pC0012	JFP55_pG0003	JFP838_pD0012	JFP838_pB0035	Hypothetical protein
JFP55_pF0012	JFP838_pC0013	JFP55_pG0002	JFP838_pD0011	JFP838_pB0036	PemK growth inhibitor
JFP55_pF0015	JFP838_pC0019	-	JFP838_pD0008	JFP838_pB0039	Hypothetical protein
JFP55_pF0017	JFP838_pC0021	-	JFP838_pD0007	JFP838_pB0041	Sortase
JFP55_pF0021	JFP838_pC0022	-	JFP838_pD0006	JFP838_pB0042	Hypothetical protein
JFP55_pF0022	JFP838_pC0023	-	JFP838_pD0005	JFP838_pB0043	Hypothetical protein
JFP55_pF0023	JFP838_pC0024	-	JFP838_pD0004	JFP838_pB0044	DNA adenine-specific methyltransferase
JFP55_pF0024	JFP838_pC0025	-	JFP838_pD0003	JFP838_pB0045	Hypothetical protein
JFP55_pF0026	JFP838_pC0027	-	JFP838_pD0001	JFP838_pB0047	Tyrosine site-specific recombinase
JFP55_pF0027	JFP838_pC0028	-	JFP838_pD0059	JFP838_pB0048	TcpA
JFP55_pF0031	JFP838_pC0029	-	JFP838_pD0057	JFP838_pB0050	TcpC
JFP55_pF0032	JFP838_pC0030	-	JFP838_pD0056	JFP838_pB0053	TcpD
JFP55_pF0033	JFP838_pC0031	-	JFP838_pD0055	JFP838_pB0054	TcpE
JFP55_pF0034	JFP838_pC0033	-	JFP838_pD0054	JFP838_pB0056	TcpF
JFP55_pF0035	JFP838_pC0037	JFP55_pG0044	JFP838_pD0053	JFP838_pB0057	TcpG
JFP55_pF0036	JFP838_pC0038	JFP55_pG0043	JFP838_pD0052	JFP838_pB0058	TcpH
JFP55_pF0037	JFP838_pC0039	JFP55_pG0042	JFP838_pD0051	JFP838_pB0059	TcpI
JFP55_pF0038	JFP838_pC0040	JFP55_pG0041	JFP838_pD0050	JFP838_pB0060	TcpJ

Apart from the conserved backbone region, comparative analysis revealed the existence of a unique ~35 kb pathogenicity locus in both NetF/NetE toxins encoding plasmids, which we designate “NetF pathogenicity locus”. This locus encoded two putative beta-channel pore-forming toxins, NetF and NetE, and other predicted proteins, which possibly contribute to the pathogenesis of *netF*-positive *C*. *perfringens* associated disease (Table 3).

**Table 3:** Summary of predicted genes in the NetF pathogenicity locus.

Locus_Tag	Length (aa)	Predicted Product	Hit Description	E value	%Identity	Superfamily/Domain
JFP838_pC0049JFP55_pF0046	391	Putative internalin A	Internalin A (*C*. *perfringens*)	2.00E-86	187/395 (47%)	LRR_RI; LRR_8; LRR_4
JFP838_pC0050JFP55_pF0047	214	Transposase	Transposase (*C*. *sordellii*)	5.00E-100	149/161 (93%)	Rve superfamily
JFP838_pC0051JFP55_pF0048	101	Transposase	Transposase (*C*. *sordellii*)	4.00E-51	90/102 (88%)	HTH superfamily
JFP838_pC0052JFP55_pF0066	57	Hypothetical protein	Hypothetical protein (*C*. *perfringens*)	9.00E-21	46/57 (81%)	-
JFP838_pC0053JFP55_pF0065	322	NetE	NetE (*C*. *perfringens*)	0	322/322 (100%)	Leukocidin
JFP838_pC0054JFP55_pF0064	259	Hypothetical protein	Gluconolactonase (*S*. *tolypothrichoides*)	0.92	66/268 (25%)	-
JFP838_pC0055JFP55_pF0063	142	Hypothetical protein	Hypothetical protein (*C*. *perfringens*)	2.00E-58	95/136 (70%)	-
JFP838_pC0056JFP55_pF0062	85	Hypothetical protein	Hypothetical protein (*C*. *perfringens*)	5.00E-33	61/86 (71%)	-
JFP838_pC0057JFP55_pF0061	175	Signal peptidase	Signal peptidase (*C*. *perfringens*)	3.00E-95	146/175 (83%)	Peptidase_S24_S26
JFP838_pC0058JFP55_pF0060	58	Hypothetical protein	Hypothetical protein (*C*. *perfringens*)	2.00E-17	39/45 (87%)	-
JFP838_pC0059JFP55_pF0059	95	Hypothetical protein	Hypothetical protein (*A*. *aromaticivorans*)	0.8	19/50 (38%)	-
JFP838_pC0060JFP55_pF0058	312	Hypothetical protein	Sugar-binding protein (*C*. *perfringens*)	0	297/312 (95%)	Bacuni_01323
JFP838_pC0061JFP55_pF0057	79	Hypothetical protein	Hypothetical protein (*C*. *perfringens*)	3.00E-45	76/78 (97%)	-
JFP838_pC0062JFP55_pF0056	300	Hypothetical protein	Hypothetical protein (*C*. *perfringens*)	0	286/300 (95%)	HTH_Hin-like superfamily
JFP838_pC0063JFP55_pF0055	135	Hypothetical protein	Hypothetical protein (*C*. *perfringens*)	3.00E-90	133/135 (99%)	-
JFP838_pC0064JFP55_pF0054	293	Transposase	Transposase (*C*. *perfringens*)	0	286/293 (98%)	PDDEXK_2
JFP838_pC0065JFP55_pF0053	308	Hypothetical protein	Hypothetical protein (*C*. *perfringens*)	0	302/308 (98%)	-
JFP838_pC0066JFP55_pF0052	55	Hypothetical protein	CHC2 zinc finger domain protein, partial (*C*. *perfringens*)	2.00E-05	21/40 (53%)	-
JFP838_pC0067JFP55_pF0051	40	Hypothetical protein	Hypothetical protein (*C*. *perfringens*)	0.013	20/22 (91%)	-
JFP838_pC0068JFP55_pF0050	641	Histidine kinase	Histidine kinase, partial (*C*. *perfringens*)	0	483/512 (94%)	HisKA superfamily
JFP838_pC0069JFP55_pF0049	305	NetF	NetF (*C*. *perfringens*)	0	305/305 (100%)	Leukocidin
JFP838_pC0070JFP55_pF0067	37	Putative transposase	Transposase (*C*. *sordellii*)	4.6	15/34 (44%)	-
JFP838_pC0071JFP55_pF0068	140	Transposase	Transposase (*C*. *sordellii*)	9.00E-40	75/85 (88%)	Rve superfamily
JFP838_pC0072JFP55_pF0069	41	Transposase	Transposase (*C*. *sordellii*)	1.00E-17	39/41 (95%)	Rve_3 superfamily
JFP838_pC0073JFP55_pF0070	664	Cell wall anchor protein	Cell wall anchor (*C*. *perfringens*)	0	603/664 (91%)	Cna_peptidase
JFP838_pC0074JFP55_pF0071	519	Cell wall surface protein	Surface protein (*C*. *perfringens*)	0	467/518 (90%)	Cna_B
JFP838_pC0075JFP55_pF0072	266	Sortase	Sortase (*C*. *perfringens*)	6.00E-143	235/265 (89%)	Sortase family
JFP838_pC0076JFP55_pF0073	136	Hypothetical protein	Hypothetical protein (*C*. *perfringens*)	4.00E-72	107/136 (79%)	-
JFP838_pC0077JFP55_pF0074	37	Hypothetical protein	Transposase, partial (*C*. *perfringens*)	3.3	17/25 (68%)	-
JFP838_pC0078JFP55_pF0075	149	Hypothetical protein	Hypothetical protein (*C*. *perfringens*)	8.00E-40	71/141 (50%)	-
JFP838_pC0079JFP55_pF0076	247	Endonuclease	Endonuclease (*Acidaminococcus* sp.)	1.00E-95	134/191 (70%)	NUC superfamily
JFP838_pC0080JFP55_pF0077	139	Hypothetical protein	Hypothetical protein (*C*. *celatum*)	2.00E-51	79/138 (57%)	-
JFP838_pC0081JFP55_pF0078	220	Resolvase	Resolvase (*C*. *perfringens*)	1.00E-148	210/217 (97%)	Ser_recombinase superfamily; PinE
JFP838_pC0082JFP55_pF0079	403	Hypothetical protein	Hypothetical protein (*Vibrio* sp.)	4.00E-20	71/246 (29%)	-

#### Features of the NetF Pathogenicity Locus

This locus consists of 34 CDSs (JFP55_pF0046—JFP55_pF0079, JFP838_pC0049—JFP838_pC0082), 18 of which were determined to be hypothetical protein-coding genes. At the 5’ end of the NetF pathogenicity locus, an internalin A-like protein was located with 30%-47% amino acid identity, respectively, with internalin-A of *Listeria monocytogenes* (GenBank CAC20628) and the putative internalin-A protein (GenBank YP_007079045) previously described on the NetB pathogenicity locus (NELoc-1). Two likely cell surface adhesion-encoding genes were found clustered near the 3’ end of the NetF locus (JFP55_pF0070 –JFP55_pF0071, JFP838_pC0073 –JFP838_pC0074). These proteins contained a Cna-like B-region collagen-binding protein domain, and a gene encoding a sortase enzyme was located immediately downstream. This region shares ~69% amino acid similarity to the group of surface proteins and sortase found on NELoc-1 of *netB*-positive *C*. *perfringens* strains (pNetB-NE10_49–53).

When compared to pJFP55F, there was a ~20 kb genomic inversion in the NetF pathogenicity locus of pJFP838C plasmid, which harboured both NetE and NetF toxin-encoding genes. Analysis of the genomic inversion indicated long (988 bp) and nearly perfect inverted repeat sequences near its termini (40133–41120, 59481–60477). In addition, the inversion was confirmed by PCR and by sequencing of each amplicon (data not shown). The presence of two transposases at the 5’ end of the inverted region (JFP838_pC0050, JFP838_pC0051) with 93%-88% identity to transposases of *Clostridium sordellii*, as well as three transposases (JFP838_pC0070, JFP838_pC0071, JFP838_pC0072) at the 3’ end of the inverted region with 95%-88% similarity to *C*. *sordellii* transposases suggests that this region of the NetF pathogenicity locus may have originated from a mobile element.

#### CPE/CPB2 Toxins-Encoding Plasmids

The second large *tcp*-conjugative family plasmids shared by the two *netF*-positive strains were designated pJFP55G and pJFP838D. They carried the *cpe* and atypical *cpb2* genes and were 36,664 bp and 48,597 bp in size, with an average G+C content of 26.2% and 26.6%, respectively. Plasmid pJFP55G is an incomplete sequence.

**Fig 2:**
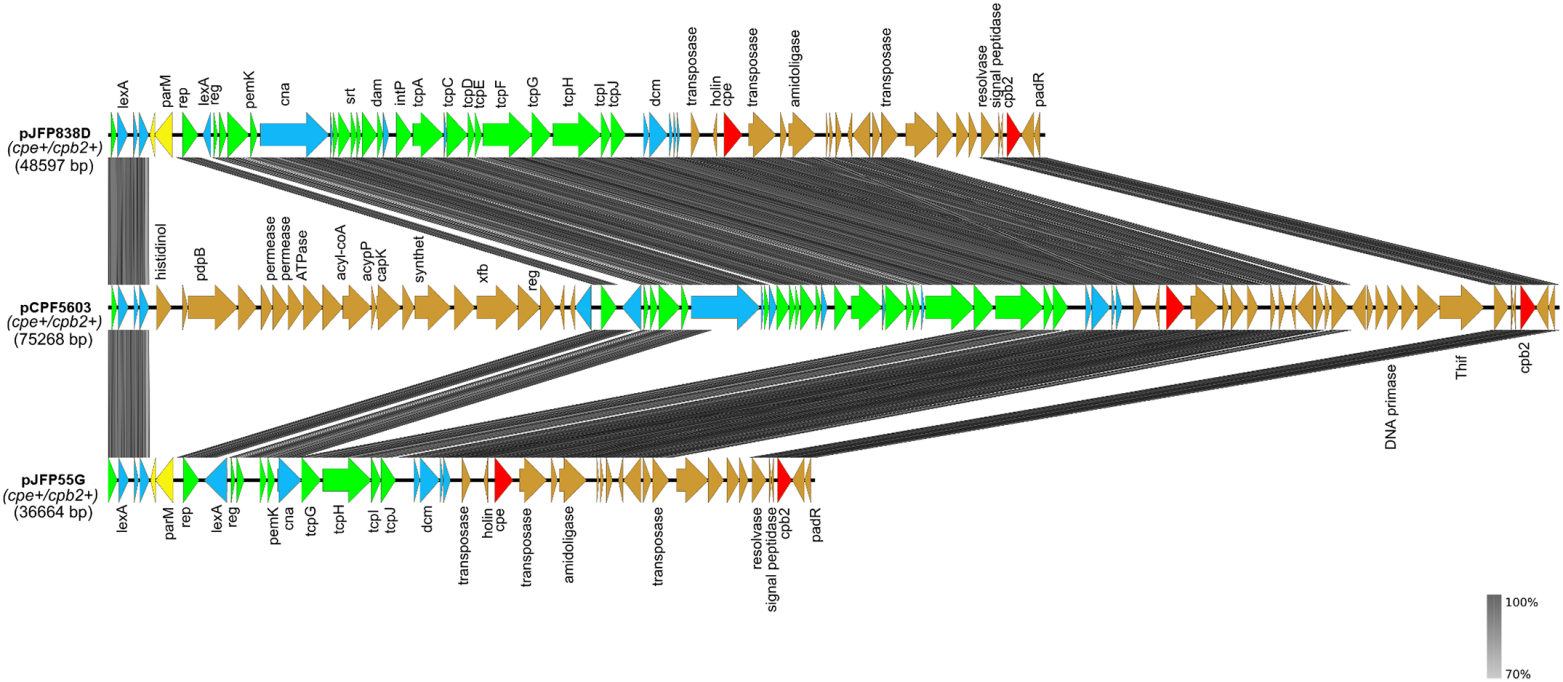
Comparative analysis of three CPE/CPB2 toxins-encoding plasmids. Grey vertical blocks indicate the shared similarity regions according to TBLASTX identity. The coloured arrows represent CDSs; CDS arrows shown are as follows: green arrows, the conserved backbone CDSs shared by all *C*. *perfringens* conjugative plasmids; blue arrows, the other conserved CDSs shared by these three plasmids; yellow arrows, unique genes on each plasmid; red arrows, toxin genes; brown arrows, unique pathogenicity locus of each plasmid. The arrows with no name are hypothetical proteins. The image was generated using Easyfig.

Plasmids pJFP55G and pJFP838D encoded 45 and 59 protein-coding sequences, respectively, and 49% of the predicted proteins were of unknown function (S1 and S2 Tables). Comparative analyses revealed that pJFP838D contains the common and conserved backbone region of *C*. *perfringens* conjugative plasmids, whereas 12/22 of conserved genes (such as *tcpACDEF*, *dam*, and tyrosine recombinase) were not found in the partial sequence of pJFP55G (Table 2). Further sequencing efforts would be required to identify the missing piece of pJFP55G compared to pJFP838D. However, pJFP55G and pJFP838D are almost identical and highly similar (98% sequence identity in 87% of plasmid length) to a ~75 kb *cpe* and *cpb2*-carrying plasmid, pCPF5603, in the type A enterotoxigenic *C*. *perfringens* F5603 strain (Fig 2).

#### Putative Bacteriocin-Encoding Plasmids

The plasmids pJFP55K and pJFP838E consist of circular DNA spanning 14,060 bp and 14,657 bp with an average G+C content 26.97% and 27.66%, respectively. These small plasmids are highly similar (99% identity at DNA level) and also (95% nucleotide identity in 70% sequence length) to the ~12 kb plasmid pCP8533S12 (GenBank AB736082) identified in type B *C*. *perfringens* strain NCTC 8533.

The annotation revealed the presence of 17 CDSs in each plasmid. A putative function could not be determined for 40% of these CDSs, (S3 and S4 Tables). Apart from the replication and maintenance related genes, pJFP55K and pJFP838E plasmids contain a cluster of genes encoding a putative efflux transporter (JFP55_pK0006, JFP838_pE0006), bacteriocin ABC transporter (JFP55_pK0007, JFP838_pE0007), and two putative bacteriocin genes (JFP55_pK0008-JFP55_pK0009, JFP838_pE0008- JFP838_pE0009).

### Plasmids unique to JFP55

Plasmid sequence analysis and comparison revealed that the JFP55 genome contains two large unique plasmids (pJFP55H, pJFP55J) absent from JFP838.

#### pJFP55H Features

pJFP55H is a ~58.5 kb circular plasmid with an average G+C content 24.72% encoding 71 CDSs. Sixty (86%) were determined to be hypothetical protein-coding genes (S5 Table). Comparative analysis showed that ~21 kb of pJFP55H (37% of plasmid length) has 74% sequence homology with a ~55 kb CPB2-encoding plasmid, pCP13, found in a type A *C*. *perfringens* that causes gas gangrene (Strain 13). Of 17 CDSs found on this homologous region, six encode known-functional proteins including a cell wall-binding protein (JFP55_pH0014), DNA topoisomerase (JFP55_pH0020), a membrane protein (JFP55_pH0028), a conjugation protein (JFP55_pH0029), a sortase (JFP55_pH0037), and a collagen adhesion protein (JFP55_pH0047). Plasmid pJFP55H also harbors two putative conjugation proteins, JFP55_pH0029 and JFP55_pH0031, which have conserved TraG_VirD4 domains.

#### pJFP55J Features

The plasmid pJFP55J consists of circular DNA of 42,209 bp with an average G+C content 24.56%. This plasmid contained 41CDSs of which 41% (17 out of 41) could not be assigned a putative function (S6 Table). pJFP55J has 27 kb (~65% of plasmid length) in common with the bacteriocin-encoding plasmid pBCNF5603 (GenBank NC_006872) in the type A enterotoxigenic *C*. *perfringens* F5603 strain, and includes a bacteriocin BCN5-like gene. The average level of DNA-DNA homology between the common regions was 99%.

### Plasmids unique to JFP838

Plasmid sequence analysis indicated that the JFP838 genome contains two large unique plasmids, pJFP838A and pJFP838B, absent from JFP55.

#### pJFP838A Features

pJFP838A is a ~404.5 kb circular plasmid with an average G+C content 24.74% encoding 490 CDSs and 10 tRNAs. Three hundred and forty-four (70.2%) were determined to be hypothetical protein-coding genes. Sequence analysis with BLASTN indicated that this plasmid is a unique mega-plasmid with virtually no homology to other clostridia sequences in GenBank. The highest homology is <1% of its sequence length (95% identity at DNA level) with a ~106 kb plasmid pCS1 (GenBank LN681235) identified in *C*. *sordellii* strain JGS6382 and 2% with the chromosome of *C*. *perfringens* ATCC13124 (92% identity at DNA level). No genes with apparent relationship to direct virulence functions were identified.

#### pJFP838B Features

pJFP838B is a ~67 kb member of the *tcp*-conjugative family of plasmids, with an average G+C content 26.34% encoding 74 CDSs. Thirty-nine of these (52.7%) were determined to be hypothetical protein-coding genes. BLASTN alignment showed that 56% of the pJFP838B sequence length has 99% similarity to the pCPF5603 plasmid (CPE/CPB2-encoding plasmid). Like the plasmids (pJFP55F, pJFP55G, pJFP838C, pJFP838D) described above, pJFP838B harbored all the conserved *tcp*-family backbone genes (Table 2). Comparative analysis indicated the presence of a unique ~31 kb pathogenicity locus in pJFP838B plasmid, which we designated the “NetG pathogenicity locus”. This locus encodes NetG, a putative beta-sheet pore-forming toxin, as well as 33 additional predicted proteins, 19 with unknown functions (Table 4 and Fig 1).

**Table 4:** Summary of predicted genes in NetG locus.

Locus_Tag	Length (aa)	Predicted Product	Hit Description	E value	%Identity	Superfamily/Domain
JFP838_pB0001	55	Hypothetical protein	Resolvase (*C*. *perfringens*)	1.00E-10	38/74 (51%)	-
JFP838_pB0002	149	Hypothetical protein	Hypothetical protein (*C*. *perfringens*)	3.00E-36	64/142 (45%)	Bacteriocin_Iid
JFP838_pB0003	142	Hypothetical protein	Hypothetical protein (*C*. *perfringens*)	3.00E-53	88/136 (65%)	-
JFP838_pB0004	98	Hypothetical protein	Transposase (*C*. *baratii*)	1.00E-37	65/97 (67%)	-
JFP838_pB0005	77	Hypothetical protein	Transposase (*C*. *baratii*)	2.00E-32	56/77 (73%)	DUF772
JFP838_pB0006	40	Hypothetical protein	Hypothetical protein (*C*. *perfringens*)	5.00E-07	23/29 (79%)	-
JFP838_pB0007	56	Hypothetical protein	Transposase (*C*. *perfringens*)	4.00E-17	42/52 (81%)	PDDEXK_2
JFP838_pB0008	306	NetG	NetG (*C*. *perfringens*)	0	306/306 (100%)	Leukocidin
JFP838_pB0009	142	Hypothetical protein	Hypothetical protein (*C*. *perfringens*)	1.00E-46	75/136 (55%)	Oxysterol_BP
JFP838_pB0010	932	Hypothetical protein	Hypothetical protein (*T*. *nexilis*)	3.00E-91	220/771(29%)	PTZ00449
JFP838_pB0011	1564	Antigenic protein NP1	Antigenic protein NP1 (*C*. *perfringens*)	0	1075/1231(87%)	M60-like superfamily; FA58C; Big_3_3
JFP838_pB0012	133	Transposase	Transposase (*C*. *pasteurianum*)	2.00E-74	109/132 (83%)	Y1_Tnp
JFP838_pB0013	73	Transposase	Transposase (*E*. *acidaminophilum*)	2.00E-33	53/73 (73%)	HTH-orfB_IS605
JFP838_pB0014	74	Transposase	Transposase (*C*. *botulinum*)	4.00E-32	59/74 (80%)	OrfB_IS605 superfamily; InsQ
JFP838_pB0015	44	Transposase	Transposase, partial (*C*. *botulinum*)	3.00E-19	38/43 (88%)	orfB_Zn-ribbon; InsQ
JFP838_pB0016	136	Hypothetical protein	Hypothetical protein (*C*. *perfringens*)	8.00E-74	108/136 (79%)	-
JFP838_pB0017	175	Signal peptidase	Signal peptidase (*C*. *perfringens*)	7.00E-90	143/175 (82%)	SigPep_I_bact
JFP838_pB0018	141	Transposase	Transposase (*C*. *perfringens*)	2.00E-78	117/137 (85%)	Transposase_mut
JFP838_pB0019	37	Hypothetical protein	Transposase (*C*. *perfringens*)	4.00E-15	36/37 (97%)	
JFP838_pB0020	234	Mitogenic protein	Mitogen (*S*. *dysgalactiae*)	6.00E-12	63/193 (33%)	Stap_Strp_Tox_C
JFP838_pB0021	49	Hypothetical protein	Transposase (*C*. *tyrobutyricum*)	4.00E-06	28/43 (65%)	-
JFP838_pB0022	312	Hypothetical protein	Sugar-binding protein (*C*. *perfringens*)	0	300/312 (96%)	Bacuni_01323_like
JFP838_pB0023	125	Transposase	Transposase (*C*. *perfringens*)	2.00E-54	96/156 (62%)	rve_3 superfamily
JFP838_pB0067	138	Transposase	TnpA (*B*. *thermotolerans*)	2.00E-36	65/131 (50%)	DUF4158
JFP838_pB0068	203	Transposase	Tn3 (*S*. *agalactiae*)	2.00E-99	159/176 (90%)	-
JFP838_pB0069	62	Hypothetical protein	Transposase (*O*. *iheyensis*)	2.00E-25	45/62 (73%)	DDE_Tnp_Tn3
JFP838_pB0070	298	TnpA transposase	Transposase (*C*. *perfringens*)	0	288/302 (95%)	DDE_Tnp_Tn3
JFP838_pB0071	275	Hypothetical protein	Hypothetical protein (*C*. *sordellii*)	1.00E-53	106/286 (37%)	-
JFP838_pB0072	90	Transposase	Transposase (*C*. *perfringens*)	3.00E-23	48/75 (64%)	PHA02517
JFP838_pB0073	133	Hypothetical protein	Hypothetical protein (*C*. *perfringens*)	3.00E-86	126/133 (95%)	-
JFP838_pB0074	103	Hypothetical protein	Hypothetical protein (*C*. *perfringens*)	4.00E-64	101/103 (98%)	-
JFP838_pB0075	300	Hypothetical protein	Hypothetical protein (*C*. *perfringens*)	0	297/300 (99%)	HTH_Hin-like superfamily
JFP838_pB0076	135	Hypothetical protein	Hypothetical protein (*C*. *perfringens*)	2.00E-90	134/135 (99%)	-
JFP838_pB0077	296	Transposase	Transposase (*C*. *perfringens*)	0	287/291(99%)	PDDEXK_2

Analyses of the NetG pathogenicity locus revealed a predicted coding sequence (JFP838_pB0011) that encodes the antigenic protein NP1. This shares 87% nucleotide similarity to a putative antigenic protein (GenBank EDT70736) found in sequences of *C*. *perfringens* strain JGS1721 (type D from sheep enteritis). Another gene was predicted to encode a mitogenic protein (JFP838_pB0020). The predicted protein shares 33% identity (E value: 1e-10) with the pyrogenic exotoxin SpeK of *Streptococcus pyogenes* (GenBank AKZ50461).

### Identification of VirR Boxes in *tcp*-Conjugative Plasmids

The VirR/VirS two-component regulatory system controls the expression of several virulence factors in *C*. *perfringens*, such as *cpa*, *cpb*, *colA*, *netB*, and *pfoA* [29,30]. Analysis of *tcp*-conjugative plasmids found in JFP55 and JFP838 revealed the presence of putative VirR boxes in the promoter region of several genes on the NetF and NetG pathogenicity loci, but not on CPE-bearing plasmids. A summary of the potential VirR/VirS-regulated genes is given in Table 5.

**Table 5:** Presence of VirR boxes in *tcp*-conjugative plasmids.

Plasmid	Locus_Tag	Product of gene	bp to start	VirR-box sequence
pJFP55F(*netF*/*netE*+)	JFP55_pF0065	NetE	95	**CCA**g**TT**atgcaagaattttaa**CCAGTT**ata**C**c
	JFP55_pF0075	Hypothetical protein	460	**CCA**g**TT**tagtataaaatatga**CCAGTT**aag**CA**
	JFP55_pF0077	Hypothetical protein	130	**CCA**a**TT**ttgcattatttttga**CCAGTT**tta**CA**
pJFP838C(*netF*/*netE*+)	JFP838_pC0069	NetE	95	**CCA**g**TT**atgcaagaattttaa**CCAGTT**ata**C**c
	JFP838_pC0078	Hypothetical protein	491	**CCA**g**TT**tagtataaaatatga**CCAGTT**aag**CA**
	JFP838_pC0080	Hypothetical protein	130	**CCA**a**TT**ttgcattatttttga**CCAGTT**tta**CA**
pJFP838B(*netG*+)	JFP838_pB0002	Hypothetical protein	78	**CCA**g**TT**ttgtatgaaatatga**CCAGTT**aaa**CA**
	JFP838_pB0008	NetG	456	**CCA**g**TT**atgtatatattttga**CCAGTT**tta**CA**
	JFP838_pB0009	Hypothetical protein	323	**CCA**g**TT**atgtatgaaatttgc**CCAGTT**atg**CA**
	JFP838_pB0025	Hypothetical protein	674	**CCA**g**TT**tggttagatatttga**CCAGTT**ctg**CA**
Consensus VirR box [31] bolded				**CCA**n**TT**nnnnnnnnnnnnnnn**CCAGTT**nnn**CA**

### Chromosome Analysis of Two *netF*-positive *C*. *perfringens*

**Fig 3:**
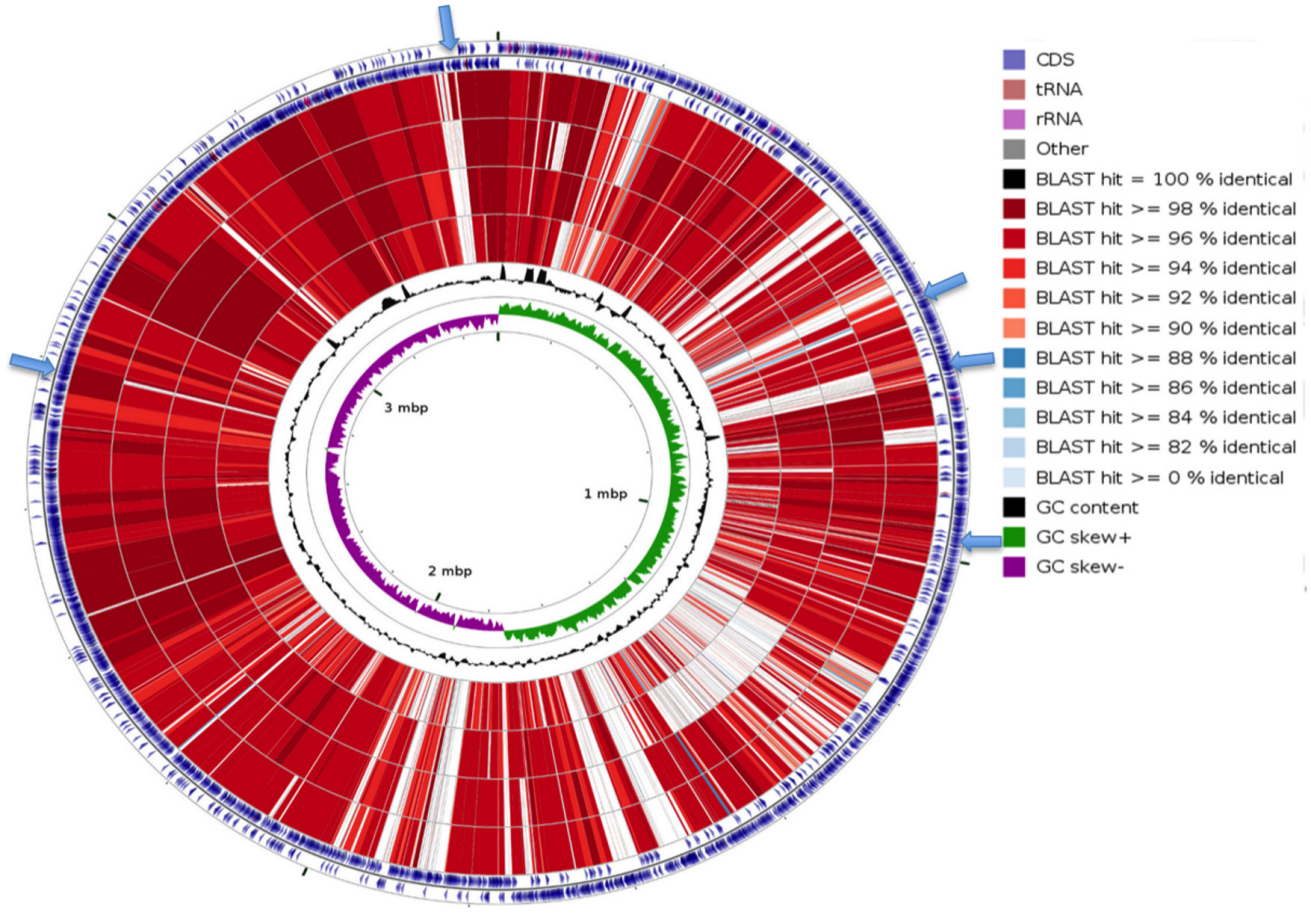
CCT map comparing the chromosomes of two *netF*-positive *C*. *perfringens* with three complete *C*. *perfringens* genomes available in NCBI. Starting from the outermost ring the feature rings depict: 1. Forward strand coding sequence of JFP838; 2. Reverse strand coding sequence of JFP838; 3. JFP55; 4. ATCC13124; 5. Strain 13; 6. SM101. The last two rings display the GC content and GC skew. The blue arrows represent some of the unique regions shared by two *netF*+ *C*. *perfringens* strains.

A summary of the general features of JFP838 and JFP55 chromosomes is presented in Table 6. Visual comparative analysis of the two *netF*-positive *C*. *perfringens* genomes with the three available complete *C*. *perfringens* chromosomes in NCBI using the CGview comparison tool [32] showed considerable genomic diversity among the finished *C*. *perfringens* sequences (Fig 3). In terms of chromosome size, JFP55 and JFP838 are slightly larger than the three other closed chromosomes and carry a number of unique regions (Fig 3).

**Table 6:** Summary of key features of chromosome of two *netF*-positive *C*. *perfringens* strains.

Strain	Source/Associated disease	Size (Mb)	G+C%	Genes	Proteins	rRNAs	tRNAs	Pseudogenes
JFP838	Canine hemorrhagic gastroenteritis	3.53	28.37	3202	3014	30	92	65
JFP55	Foal necrotizing enteritis	3.34	28.37	3033	2825	30	94	83

In addition, PHAST analysis revealed that the chromosome of JFP55 contains an intact ~55 kb prophage related to *Clostridium* phage vB_CpeS-CP51 (GenBank NC_021325) whereas no complete phage region was observed in JFP838. However, the JFP838 chromosome contains four incomplete or questionable integrated phage segments (total ~81 kb).

### Identification of Unique Nucleotide Sequence in the *netF*-positive *C*. *perfringens* using PanSeq

Panseq analysis was used to identify chromosomal sequences shared between JFP55 and JFP838 but not present ATCC13124, SM101 and Strain 13. A summary of the features and CDSs of JFP838 and JFP55 identified by PanSeq in the unique regions not found in the three published genomes of *netF*-negative strains is presented in Table 7, S7 and S8 Tables.

**Table 7:** General features of unique regions identified by PanSeq.

Strain	#Unique regions	#CDSs	#Hypothetical proteins	#Regulatory proteins	#Pseudogenes	#Recombination associated proteins	Phage-related region	Plasmid-related region	Total size (kb)
JFP55	48	240	163	9	25	13	5	2	247
JFP838	81	413	237	16	35	12	9	1	430

These chromosomal unique regions encoded 240 and 413 genes in JFP55 and JFP838, respectively. Five unique regions of JFP55 (JFP55_UR3-UR7) were prophage-related regions, ~23 kb in size. Prophage-related regions were also identified in 11% of JFP838 unique regions (9/81 unique regions; ~83 kb) (JFP838_UR34-40, UR57 and UR59).

BLAST alignments between the unique regions of JFP55 and JFP838 indicated that 16 (~69 kb) were common to both *netF*-positive strains. The features of those unique to the two *netF*-positive strains in comparison to the three reference genomes but shared between JFP55 and JFP838 are presented in S9 Table.

The average extent of DNA-DNA homology between these common regions was 94%. These regions contained 69 unique CDSs of which 58% (40 of 69) could not be assigned a putative function. No shared virulence genes unique to the two *netF*-positive chromosomes were identified. Interestingly, five of these shared regions were part of a larger region, split into these five small sub-regions. For instance, the unique region, JFP838_UR22 (a 38 kb plasmid-related region), was broken into five shared closely located regions (SUR_4-SUR_8).

## Discussion

The current typing system for *C*. *perfringens* is inadequate, in particular for type A isolates. In recent years, it has become clear that there are important and distinct subsets of “type A” *C*. *perfringens*. These include, for example, CPE enterotoxin-producing strains associated with food poisoning in humans [33] or with antibiotic-associated diarrhea in humans [34]. In addition, there have been major discoveries in advancing understanding of the diversity of pathotypes present among “type A” *C*. *perfringens* including the description of the beta-sheet pore-forming toxin NetB in necrotic enteritis (NE) of chickens [35], and the recognition of novel pathogenicity loci in the conjugative plasmids of NE isolates [11], as well as the clonality of NE isolates [36]. Most recently, the pathotype diversity has been highlighted by the discovery of the large conjugative plasmid-encoded toxin NetF, its association with canine hemorrhagic gastroenteritis and foal necrotizing enteritis, and the common clonal lineage of these isolates [6]. It is clear that the traditional toxinotyping scheme requires modifications to include these new findings, and to adapt to the diversity of distinct enteric disease caused by this bacterium.

The current study provides the first complete genome sequences of two *netF*-positive *C*. *perfringens* strains, JFP55 and JFP838, greater insight into the potential pathogenesis of *netF*-positive *C*. *perfringens* strains associated disease, and expands our understanding of both genomic diversity and of pathogenicity loci in *tcp*-conjugative plasmids in this important enteric pathogen. Considerable work remains to be done to understand the contributions of the novel genes and loci identified in this study. In relation to pathogenic *C*. *perfringens*, we define a pathogenicity locus as a genetic region unique to a particular pathotype that contains one or more virulence genes, as well as mobility-associated genes. A previous paper [11] described regions unique to the *netB*-pathotype as pathogenicity loci (PaLoc).

We found that these NetF-producing *C*. *perfringens* strains harbor three plasmids in common, including two *tcp*-conjugative plasmids, which encode *netF*/*netE* and *cpe*/*cpb2* toxins genes, and a putative bacteriocin-encoding plasmid. This finding suggests that the key event in the evolution of *netF*-positive *C*. *perfringens*-associated foal and canine necrotizing enteritis was likely acquisition of NetF/NetE plasmid, since these are common to both strains and are defining characteristic of these strains. The hypothesis of a key evolutionary event is further supported by the previous finding that the presence of NetF is crucial for producing cytotoxicity *in vitro* [6]. The 20 kb inversion of the region of the NetF pathogenicity locus containing the *netE* and *netF* genes suggests the mechanism by which this pathogenicity locus added the critically important *netF* toxin gene [6], since this region is flanked by a large inverted repeat. Acquisition of this region was likely an important event in the evolution of this virulence plasmid and of this *C*. *perfringens* pathovar. An interesting trait of the *netF*-positive strains is that they always contain a CPE-bearing plasmid [6]. The consistent presence of *cpe* plasmid in these strains suggests that the ancestral strain also possessed this plasmid, or acquired it early in stages of expansion of this lineage, and also that CPE production may be important in the pathogenesis of disease caused by *netF*-positive strains [6]. Recently, Uzal and others [37] have demonstrated a synergistic effect of CPB and CPE of a type C human enteritis necroticans strain in producing histological damage and fluid accumulation in rabbit intestinal loops. However, further research is required to identify the exact role of NetE and CPE in the pathogenesis of *netF*-positive *C*. *perfringens* infections.

A small bacteriocin-carrying plasmid also co-exists with NetF/NetE- and CPE/CPB2-bearing plasmids in the sequenced strains. It has been shown that the production of bacteriocins is a common feature of *C*. *perfringens*, and might contribute to enhancement of intestinal colonization by overcoming the normal intestinal flora [8,38,39]. The presence of the closely related bacteriocin plasmid in both NetF-producing strains suggests its importance in this lineage.

Apart from the common plasmids in both *netF*-positive strains, the genome of each strain contains two unique plasmids. One unique plasmid of interest is the mega-plasmid, pJFP838A. Although mega-plasmids are a common feature in some clostridal species, such as neurotoxigenic *C*. *butyricum* and *C*. *botulinum* [40,41], they have not been previously reported in *C*. *perfringens*. The variable presence of these unique plasmids in NetF-producing strains suggests that these have been acquired during evolution from the ancestral strains and may not be important in virulence.

Our group has previously shown that the variable presence of *netG* is a feature of *netF*-positive strains, since *netG* was only present in 46% (5/11) and 47% (7/15) isolates from canine hemorrhagic gastroenteritis and foal necrotizing enteritis isolates, respectively [6]. The inconsistent presence of this putative toxin gene in *netF*-positive *C*. *perfringens* strains suggests that it is likely less important in the virulence of these strains [6].

The toxin-encoding plasmids described in the current study are members of *tcp*-conjugative family plasmids. These plasmids encode the *tcp* locus, which shares minor sequence homology with Tn916 conjugative transposon family [19]. It is therefore likely that these are conjugative plasmids but we did not explore this and this still needs to be tested in conjugation experiments.

A general feature of *C*. *perfringens* toxin-carrying plasmids is the location of many toxin genes on pathogenicity loci close to the DNA cytosine-methytransferase (*dcm*) region, an insertional hot-spot for the mobile genetic elements that encode the toxin genes [9,12,22]. For instance, the gene encoding NetB is localized downstream of the conserved *dcm* region on conjugative variably-sized plasmids (80–90 kb) [9,16,35]. Lepp and others [11] identified that *netB* along with 36 additional genes are present on a large pathogenicity locus (~42 kb).

This study demonstrates for the first time that *netF* is localized on a ~35 kb plasmid-encoded pathogenicity locus in *netF*-positive strains. Although functional studies are required to demonstrate the role of the genes residing on the “NetF pathogenicity locus” in the pathogenesis of *netF*+ *C*. *perfringens*-associated enteric infections, sequence annotation and comparative analysis will assist future studies. The NetF pathogenicity locus (Table 3) consists of 34 CDSs, 18 of which could not be assigned a putative function. Interesting features of this include an internalin A-like protein, as well as, two putative cell surface adhesion proteins. The internalin family was originally identified in *Listeria monocytogenes* as cell surface proteins which mediate the bacterial adhesion and invasion [42]. In some *Clostridium* species such as *C*. *botulinum*, *C*. *perfringens* and *C*. *tetani*, cell surface proteins with homology to *L*. *monocytogenes* internalins were also identified [11,43,44]. In the NetB pathogenicity locus, a putative internalin-like protein was also found immediately upstream from *netB* gene [11]. While the role of these internalin-like proteins has not yet been fully defined, the presence of leucine-rich repeats domains suggests that they are likely involved in protein-protein interaction [45].

Other features of interest were two likely cell surface encoding genes and a sortase gene found clustered near the 3’ end of the NetF pathogenicity locus that exhibited close similarity to the group of surface proteins and sortase found on NELoc-1 of *netB*-positive *C*. *perfringens* strains. These surface proteins contained a Cna-like B-region domain, which is originally found in the *Staphylococcus aureus* collagen-binding protein where it acts as a stalk to present the ligand-binding domain of adhesion away from the bacterial cell surface [46]. Interestingly, the JFP55_pF0071 and JFP838_pC0074 proteins additionally had a fimbrial isopeptide formation D2 domain. This domain was found in the *Streptococcus pneumoniae* pilus protein, RrgB, and acts in many Gram-positive surface proteins either as pilin subunit cross-linking or cell wall attachment [47]. Further functional studies are required to elucidate the possible contributions of these proteins in bacterial attachment to the host cell surface.

Sequence analysis of the NetF pathogenicity locus also revealed the presence of VirR-boxes upstream of two hypothetical proteins (JFP55_pF0075, JFP55_pF0077 –JFP838_pC0078, JFP838_pC0080), as well as, of *netE* in both *netF*-positive strains, suggesting that these genes are co-regulated by two-component VirR/VirR regulatory system, as is *netB* [30].

The *cpe* plasmids of type A *C*. *perfringens* strains are classified into two main families: a) pCPF5603-like plasmids which are usually ~75 kb in size and harbor *cpe* and *cpb2* b) pCPF4969-like plasmids that are typically ~70 kb in size and harbor *cpe* and *bcn* (bacteriocin gene). The *cpe* gene on both types of plasmids is flanked by an upstream IS*1469* sequence. However, the IS*1151* and IS1470 sequences are found downstream of *cpe* on pCPF5603- and pCPF4969-like plasmids, respectively [48–50]. CPE/CPB2-bearing plasmids in NetF-producing strains are highly similar to pCPF5603-like plasmids, and carry the same IS sequences as pCPF5603. The CPE/CPB2 pathogenicity locus in *netF*-positive strains is ~17 kb and harbors four hypothetical proteins (JFP55_pG0020-23 –JFP838_pD0029-32) which are absent from pCPF5603. Three have conserved domains, including a trypsin-like serine protease (JFP55_pG0021, JFP838_pD0030), HNH nuclease (JFP55_pG0022, JFP838_pD0031) and ATPase (JFP55_pG0023, JFP838_pD0032). Apart from these unique genes on CPE/CPB2 pathogenicity locus of *netF*-positive strains, the rest of the locus is largely identical to that of pCPF5603. One interesting finding in both pJFP55G and pJFP838D plasmids was the presence of a holin-like protein (JFP55_pG0035, JFP838_pD0044) located immediately downstream of the enterotoxin gene. A previous study in *Clostridium difficile* has shown that the holin-like protein, TcdE, is required for export of the enterotoxins TcdA and TcdB [51]. Whether the holin-like protein found in the enterotoxin locus of both pJFP55G and pJFP838D plays a role in exporting of enterotoxin remains to be investigated.

As noted, the variable presence of *netG* is a feature of NetF-producing strains. We found that *netG* is located on a ~31 kb unique pathogenicity locus. One CDS of interest on the NetG locus, the antigenic protein NP1 (JFP838_pB0011), exhibits ~30% amino acid homology with F5/8 type C domain-containing protein, CP4_3468, found in *netB* plasmid [11]. This protein contains two domains, the peptidase M60-like superfamily (E value: 4E-36) and discoidin family domain (E value: 2E-08). The M60-like superfamily contains a zinc metallopeptidase shown to be involved in mucinase activity [52]. In addition, proteins containing discoidin domains are predicted to bind carbohydrates such as galactose [53]. An intriguing hypothesis is that this protein may be involved in mucin colonization of *C*. *perfringens*. In addition, the NetG pathogenicity locus contains VirR-boxes upstream of two hypothetical proteins (JFP838_pB0002, JFP838_pB0009), as well as, NetG (JFP838_pB0008), suggesting that these genes are likely co-regulated by two-component VirR/VirR regulatory system and therefore possibly important in virulence.

The two chromosome sequences (JFP55 and JFP838) are slightly larger than the other three completely closed *C*. *perfringens* chromosomes [7,8]. This finding suggests that these NetF-producing strains harbor chromosomal unique regions missing in the three reference strains. The novel region finder of PanSeq tool identified regions unique to each of the chromosome of JFP55 and JFP838, respectively and absent from the chromosome of three references strains. Large unique regions included complete and partial phage sequences, as well as regions likely associated with capsule formation. In addition, ~86 and 38 kb of total length of the unique regions in JFP55 and JFP8383, respectively, was plasmid related but chromosomally-integrated. These regions have some classic hallmarks of plasmid genes, such as the collagen-binding protein first identified in pCP13 by Shimizu and others [7]. Although it is well known that the enterotoxin gene (*cpe*) can move between plasmid and chromosome of *C*. *perfringens* [54,55], the integration of a large piece of plasmid DNA (~18 kb) into a *C*. *perfringens* chromosome was first described in a type A *C*. *perfringens* isolate recovered from a case of bovine abomasitis [56]. We found that only 16 unique regions were shared by two *netF*-positive *C*. *perfringens* strains. Five of these common regions formed a mosaic of plasmid-integrated segments. These five regions are adjacent and likely originate from a single integration event followed by recombination. This finding suggests that these elements were acquired early in a clonal lineage of *netF*-positive *C*. *perfringens* strains. In addition, the presence of multiple chromosomal unique regions, which are not shared by the two *netF*-positive strains suggests these strains subsequently diverged for an extended time. Further work is required to assess the significance of chromosomal regions unique to NetF-producing *C*. *perfringens* strains.

Interestingly, one of the plasmid-integrated genes in the chromosome of both NetF-producing strains was the collagen adhesin-encoding gene, SUR_4 (S9 Table), which has been suggested to facilitate colonization [7]. In addition to this chromosomally encoded adherence factor, we found three and two other collagen binding proteins on the *tcp*-conjugative plasmids of JFP838 and JFP55, respectively. The presence of this number of adhesin genes is intriguing and suggests a possible role in the intestinal colonization of *netF*-positive strains.

In summary, we found that the JFP55 and JFP838 strains, which originated from foal necrotizing enteritis and canine hemorrhagic gastroenteritis cases, share unique virulence genes on conserved pathogenicity loci found on large *tcp*-conjugative plasmids. The identification of common features for these two strains provides supportive evidence that these two *netF*-positive strains are a part of a common clonal lineage [6]. Moreover, these results provide significant insight into the potential pathogenesis basis of canine and foal necrotizing enteritis and into the evolution of virulence of *C*. *perfringens* involved in enteric disease.

## Supporting Information

S1 Table Summary of predicted genes identified by MyRast software in pJFP55G. (XLSX)

S2 Table Summary of predicted genes identified by MyRast software in pJFP838D. (XLSX)

S3 Table Summary of predicted genes identified by MyRast software in pJFP55K. (XLSX)

S4 Table Summary of predicted genes identified by MyRast software in pJFP838E. (XLSX)

S5 Table Summary of predicted genes identified by MyRast software in pJFP55H. (XLSX)

S6 Table Summary of predicted genes identified by MyRast software in pJFP55J. (XLSX)

S7 Table Unique chromosomal nucleotide sequences of JFP838 identified by PanSeq. (XLSX)

S8 Table Unique chromosomal nucleotide sequences of JFP55 identified by PanSeq. (XLSX)

S9 Table Shared unique chromosomal nucleotide sequences by two *netF*-positive *C*. *perfringens*. (XLSX)
